# NIA SBIR-FUNDED JUVENA THERAPEUTICS TRANSLATION OF POWERFUL PROTEINS INTO TRANSFORMATIVE THERAPEUTICS

**DOI:** 10.1093/geroni/igae098.0491

**Published:** 2024-12-31

**Authors:** Jeremy O’Connell

**Affiliations:** Juvena Therapeutics, Inc., Redwood City, California, United States

## Abstract

Juvena Therapeutics, which received NIA SBIR funding in 2021, was founded to restore tissue health by repairing protein signaling and intercellular communication. Juvena maps the therapeutic potential of natural secreted proteins and engineers them into biologics that restore tissue homeostasis. Juvena’s artificial intelligence-enabled platform mines thousands of proteins secreted by human stem cells, screens their potential to restore tissue homeostasis, and engineers novel biologic medicines into life-saving treatments. Juvena’s SBIR funding had multiple benefits as it enabled optimization of their lead compound, additional compound screenings, as well as the rigorous feedback that resulted from peer review. Since receiving NIA SBIR funding and showcase support, Juvena has now raised close to $60 million in venture capital funding and is rapidly advancing its efforts. Juvena’s journey is a great example of how SBIR funding can be a critical resource early in a biotech startup’s development path.

